# Taxing the Doctors: A Protest

**Published:** 1897-11

**Authors:** 


TAXING THE DOCTORS: A PROTEST.—Alas, poor old Texas! With her unlimited resources,—resources scarcely touched, and wealth undreamt of, she *feels* that she is driven to the expedient of taxing her doctors to raise revenue enough to carry on business at the old stand. The JOURNAL enters its most earnest protest against a measure so unjust, and so unnecessary. The *ad valorem* tax on real estate is only 18c. upon the $100, and the valuation is always placed at the lowest notch; lands worth in the market $5 per acre, are returned on the tax rolls at 50c. and $1 per acre. Were property owners compelled to return their holdings at anything like reasonable figures, or were 1c. on the $100 added, the increase in the revenue would be largely in excess of the total amount yielded by this unjust levy on the physicians. It carries us back to the old "reconstruction" days, when the South was paying the additional penalty of her temerity in daring to assert the right to peaceable secession; the aftermath, the "indemnity," the "spoils" which belonged to the "victor." In those days, everybody and everything in the South was taxed; every document of a business character, every bank check on one's balance in bank, had to have a "revenue" stamp on it. (If the money had been in pocket, the government would have required a stamp on the seat of one's breeches, no doubt.) Some say that a man had to put a revenue stamp on his mouth every time he kissed his wife. But that is mere hearsay; I don't believe it.

It was to be expected in those days—no one looked for justice; but now, in these piping times of peace, that a State should stoop to such meanness as to put a tax on the privilege of working for nothing—for we venture the assertion that doctors do more charity practice than they do paying practice,—or to tax the privilege of practicing medicine for pay,—is most surprising and unusual. Why not make a clean sweep of it, and tax the ministers of the gospel? They derive revenue from their calling; they must live, and few doctors do more—they derive not more than enough revenue from their work to enable them to keep body and soul together, and that, too, notwithstanding they do the hardest kind of work, even thankless work, most frequently. It is a benevolent profession and should not be taxed. Why not tax the day laborer, the mechanic, the chimney sweep? Tax everybody on their living,—on their privilege of making a bare living? The wise men of the legislature who made this wise law are so fond of putting the doctors on a level with the boot-blacks and the barbers—as will be recalled in connection with every attempt the medical profession has made to get a bill passed to protect the public from the ignorant and unscrupulous who steal the livery of the doctor to serve the devil and themselves in—let them be consistent and carry out their joke (?) and tax the boot-black too.

Seriously, the occasion calls for an expression from the medical profession of an earnest and united protest. We publish elsewhere in this issue a resolution on the subject adopted by the physicians of Navarro County, protesting against the law. We hope every medical society in Texas will meet and take similar action; and at the next session of the legislature let there be a memorial from the united profession asking for a repeal of the burdensome, and unjust, and altogether unnecessary tax. It is only $5 a year; but $5 a year to the country physician is a large sum ("with cotton at 5c. per pound"). It would pay for the TEXAS MEDICAL JOURNAL two years and a half. It will buy a real nice suit of clothes—and no end to shoes and stockings for Betty and the baby.

The Austin doctors are even worse off than their brethren; for, in addition to the State tax of $5, we pay a city and county tax of $5 a year. "*Down with the stamp act.*"

---

AND NOW COMES a man who ought to know; know better than almost any other man—and says that mad dogs are not afraid of water, and that hydrophobia is, therefore, a misnomer. Mr. Hines' article (Abstracts and Selections: "Rabies,") will be read with interest.

